# Evaluation of methylene blue restaining versus conventional hydrogen peroxide decolorization in immunohistochemical diagnosis of melanoma

**DOI:** 10.1038/s41598-025-89186-8

**Published:** 2025-02-10

**Authors:** Weisong Wan, Le Wang, Yuye Zeng, Yuchang Hu, Yufei Liu

**Affiliations:** 1 The First Clinical Medical College of China, Three Gorges University, Yichang, 443000 China; 2 Institute of Pathology, China Three Gorges University, Yichang, 443000 China; 3 Department of Pathology, Yichang Central People’s Hospital, Yichang, 443000 China

**Keywords:** Melanoma, Immunohistochemistry, Methylene blue restaining, Hydrogen peroxide bleaching, Melanin, Immunohistochemistry, Melanoma

## Abstract

This study seeks to address the challenge of melanoma identification in immunohistochemical (IHC) diagnosis, which is complicated by the similar coloration of melanin and DAB (Diaminobenzidine) staining, by introducing methylene blue counterstaining as an innovative solution. We compared the effectiveness of methylene blue counterstaining with that of the traditional hydrogen peroxide bleaching method in the diagnosis of melanoma. The study included 46 paraffin-embedded melanoma samples, and the staining efficacy for markers such as Melan A, HMB-45, PRAME, and Ki-67 was assessed via both methods. The results demonstrated that methylene blue counterstaining effectively converted the brownish-yellow melanin granules to a deep green color, significantly enhancing contrast and clarity with DAB staining. The average contrast and clarity scores for the methylene blue counterstaining method were 1.96 ± 0.21 and 1.91 ± 0.28, respectively, which were significantly greater than those of the conventional IHC group and the hydrogen peroxide bleaching group (*P* < 0.01). Furthermore, methylene blue counterstaining did not cause noticeable tissue damage or cellular morphology distortion, with tissue integrity scores comparable to those of the conventional IHC group (*P* > 0.05). Although the contrast and clarity also improved in the hydrogen peroxide bleaching group, it resulted in a significant decrease in tissue integrity (*P* < 0.01). This study is the first to apply methylene blue counterstaining in melanoma IHC analysis, demonstrating its advantages in enhancing staining quality, simplifying procedural workflows, and preserving antigenicity. This method provides a novel and effective tool for the pathological diagnosis of melanoma, potentially improving diagnostic accuracy and reliability.

## Introduction

Melanoma is a highly aggressive and lethal skin cancer that originates from melanocytes. According to statistics, approximately 232,100 new cases of melanoma were diagnosed globally in 2020, accounting for 1.7% of all newly diagnosed primary malignant tumors. Each year, approximately 55,500 individuals die from melanoma, representing 0.7% of all cancer-related deaths^1,2^. In China, although the overall incidence of melanoma remains relatively low, it is increasing at an annual rate of 3–5%, making it one of the fastest-growing malignant tumors, with approximately 20,000 new cases each year^3,4^. Early diagnosis and intervention can significantly improve patient survival rates and reduce mortality. Therefore, accurate and standardized pathological diagnosis is crucial for clinical treatment and prognosis assessment.

Immunohistochemistry (IHC) is a technique that uses specific antibodies to label specific antigens within tumor cells, providing detailed information about tumor phenotypes. In the pathological diagnosis of melanoma, IHC is widely employed to confirm the tumor’s origin and its biological characteristics. However, there are significant challenges when dealing with melanoma tissue containing a large amount of melanin. The natural color of melanin (usually brownish-yellow) is very similar to that of the DAB chromogenic product (also brownish-yellow), making it difficult to distinguish between the two under a microscope, which severely affects pathologists’ accurate interpretation of the IHC results^5^.

Traditionally, strong oxidizing agents such as potassium permanganate and hydrogen peroxide have been used to decolorize melanin. The strong oxidative effect of potassium permanganate effectively removes melanin but potentially damages the tissue structure and affects antigen strength^6^. While hydrogen peroxide is relatively easy to use, it also has similar limitations, such as causing tissue damage and requiring a longer processing time^7,8^. Additionally, some researchers have used red AP (alkaline phosphatase) chromogenic agents as alternatives; however, these agents often produce a lower positive intensity than DAB does, and the stained sections require aqueous mounting media or glycerol jelly for sealing, which limits their long-term preservation and observation^9^.

To address these limitations, this study proposes a novel solution—the methylene blue restaining method. This method uses methylene blue dye to convert the brownish-yellow melanin granules into a dark green color, thereby significantly enhancing the color contrast between melanin and the DAB chromogenic product. Methylene blue, a well-established dye, can provide clear color differentiation without significantly affecting tissue structure or antigenicity.

This study is the first to apply the methylene blue restaining method to melanoma tissue and evaluate its value in distinguishing melanin granules from DAB chromogenic products. Preliminary results indicate that this method achieves excellent efficacy in improving recognition accuracy under a microscope, providing a simple and effective solution to the shortcomings of traditional methods.

## Results

### Clinical characteristics of the patients

A total of 46 melanoma cases with melanin were identified through searches conducted within the pathology department’s case management system and pathological reviews conducted by physicians. Among these patients, 22 were male and 24 were female, with ages ranging from 26 to 73 years and an average age of 62.3 ± 12.3 years. The primary locations of occurrence included the skin of the hands and feet (29 patients), the nasal cavity (14 patients), the face (2 patients), and the conjunctiva of the eye (1 patient). 

### HE staining results

**Fig. 1:**
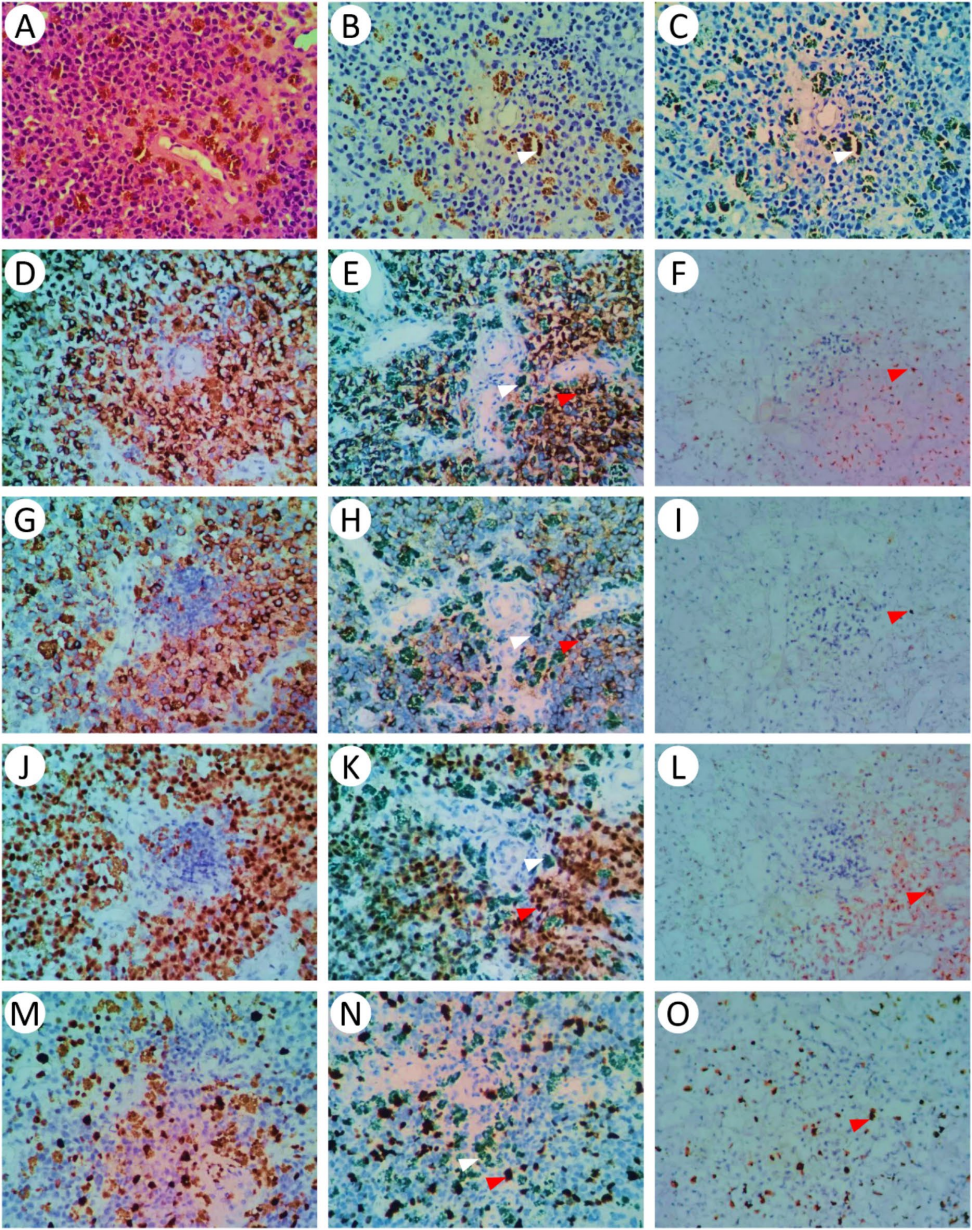
Staining results of different melanin treatment groups. (**A**): HE staining; (**B**): Routine IHC group blank control, using PBS instead of primary antibody as a control; (**C**): Methylene blue restaining group blank control, using PBS instead of primary antibody as a control; (**D**), (**G**), (**J**), and (**M**): Routine IHC group, immunohistochemical staining with Melan A, HMB-45, PRAME, and Ki-67, followed by hematoxylin counterstaining; (**E**), (**H**), (**K**), and (**N**): Methylene blue restaining group, immunohistochemical staining with Melan A, HMB-45, PRAME, and Ki-67, followed by hematoxylin staining and then methylene blue counterstaining (The white triangle symbol represents melanin, and the red triangle symbol represents DAB staining); (**F**), (**I**), (**L**), and (**O**): Hydrogen peroxide decolorization group, tissue treated with hydrogen peroxide to remove melanin, followed by immunohistochemical staining with Melan A, HMB-45, PRAME, and Ki-67, and hematoxylin counterstaining (The red triangle symbol represents DAB staining). The magnification of all the images is ×600.

HE staining revealed varying amounts of brownish-yellow melanin granules (Fig. 1A).

### IHC staining results

In the conventional IHC group, melanin granules presented a brownish‒yellow color similar to that of DAB staining (Fig. 1B, D, G, J, and M). The contrast and clarity scores for melanin and tumor cell staining were 0.09 ± 0.28 and 0.85 ± 0.36, respectively. No tissue damage or disruption of the cellular morphology was observed in the tissue sections, with an integrity score of 1.98 ± 0.15.

In the methylene blue restaining group, methylene blue restaining transformed the brownish-yellow melanin granules into a deep green hue (Fig. 1C, E, H, K, and N). Compared with the other groups, this group presented significantly higher scores for both staining contrast (1.96 ± 0.21) and clarity (1.91 ± 0.28) (*P* < 0.01). Similar to the conventional IHC group, no significant tissue damage or disruption of cellular morphology was observed, with a tissue integrity score of 1.96 ± 0.21, which was not significantly different from that of the conventional IHC group (*P* > 0.05).

**Fig. 2:**
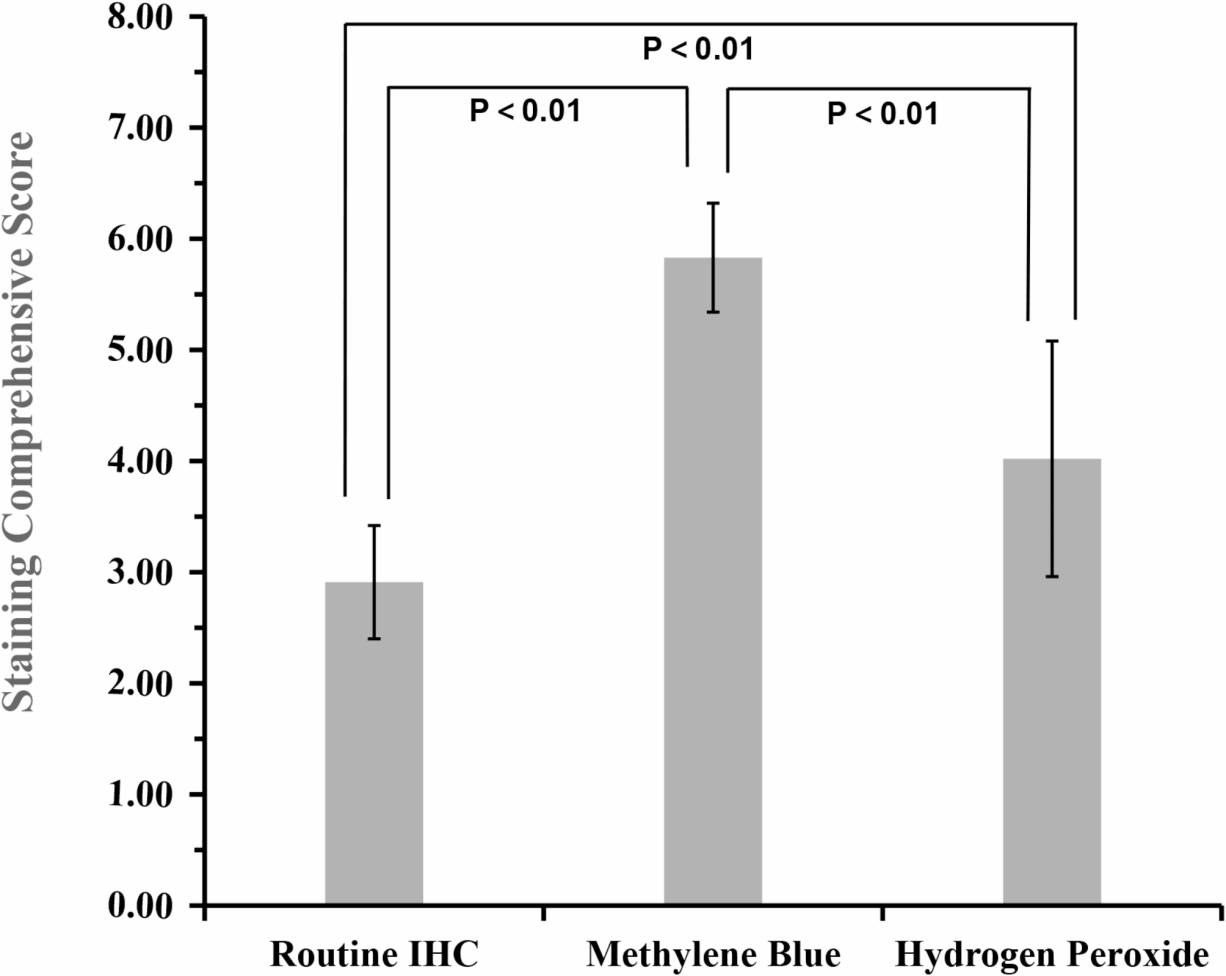
Comprehensive staining scores of the different treatment groups.

In the hydrogen peroxide decolorization group, some tissue sections in this group presented residual melanin, weakened positive IHC intensity, and exhibited tissue damage and disruption of cellular morphology, with some sections even experiencing detachment (Fig. 1F, I, L, and O). The tissue integrity score of the hydrogen peroxide decolorization group (0.43 ± 0.72) was significantly lower than that of the conventional IHC group (*P* < 0.01). However, this group had higher scores for staining contrast (1.80 ± 0.40) and clarity (1.78 ± 0.51) than did the conventional IHC group (*P* < 0.01). The comprehensive IHC staining score of different treatment groups was compared as shown in Fig. 2. Methylene blue restaining group was significantly higher than hydrogen peroxide decolorization group and conventional IHC group (*P* < 0.01).

## Discussion

Melanin, a pigment widely distributed in biological systems, particularly within the skin and hair, plays a crucial role in the diagnosis of various diseases because of its significant presence. However, in IHC staining, melanin particles often pose a challenge for accurate identification because of their brownish-yellow color, which is similar to that of the DAB chromogen^5,10^. As demonstrated in Fig. 2B, D, G, J, and M of this study, melanin granules in the conventional IHC groups presented a brown–yellow hue analogous to that of DAB staining. This color similarity results in inadequate contrast in IHC staining, potentially complicating the distinction between melanin and other tissue structures, thereby impacting the accuracy of pathological diagnosis.

To address this issue, current research, both domestically and internationally, has focused primarily on two approaches. The first approach involves the use of strong oxidizing agents for decolorization, where potent oxidants such as potassium permanganate or hydrogen peroxide are employed to bleach melanin. The core principle here is that melanin can be decolorized by breaking its phenolic rings through the oxidative action of these agents^7,10–13^. The second approach is heterochromatic staining, which uses certain chemical reagents to stain melanin, which was originally brownish-yellow in IHC, into a different color that can be easily distinguished from DAB staining^5,14–16^.

In this study, for the first time, we applied methylene blue for immunohistochemical analysis of melanoma. By evaluating the IHC staining of commonly used markers in melanoma diagnosis and assessment—namely, Melan A, HMB-45, PRAME, and Ki-67 (The first two stain the cytoplasm, while the latter two stain the nucleus.)—we demonstrated the superior application value of this technique.

Methylene blue, a low-molecular-weight biological dye, has been extensively documented for its applications in biological tracing and special staining techniques^17^. The methylene blue counterstaining method involves the application of methylene blue solution after conventional IHC staining and hematoxylin counterstaining. This approach converts brown‒yellow melanin granules to a dark green hue, creating a stark contrast with the DAB chromogen, thereby significantly enhancing the visualization of melanin granules. As illustrated in Fig. 2C, E, H, K, and N, methylene blue counterstaining remarkably improved the staining quality by transforming the brown–yellow melanin granules into a dark green color, which contrasts sharply with brown DAB staining, thereby markedly increasing the contrast and clarity of the staining. The melanin and tumor cell staining contrast scores (1.96 ± 0.21) and staining clarity scores (1.91 ± 0.28) were significantly greater in the methylene blue counterstaining group than in the other groups (*P* < 0.01). Additionally, minimal tissue damage or disruption of the cellular morphology was observed in the tissue sections, with no significant differences noted compared with those in the conventional IHC group.

One notable advantage of the methylene blue counterstaining method is its ease of use, requiring only 2–3 s for the staining process. Compared with other counterstaining agents, such as Giemsa^14^, azure blue^5^, ferrous sulfate^15^, and methyl green^16^, methylene blue counterstaining offers a significant time advantage, allowing simultaneous processing and imaging with other immunohistochemical slides. Additionally, the methylene blue counterstaining method maintains a fixed staining time, ensuring that tissues with low to high melanin content can be stained within 2–3 s. This contrasts with depigmentation methods using oxidizing agents, where the reaction time must be extended as the melanin content increases, thus significantly enhancing the experimental efficiency. Furthermore, since the entire procedure does not require strong oxidizing agents, the integrity of antigens is preserved, avoiding issues of antigen weakening or false negatives. It is crucial to strictly control the timing of methylene blue solution application and ensure thorough rinsing to prevent residual methylene blue from affecting the results. Through extensive practice, we have shown that after methylene blue counterstaining and rinsing with running water, the slides can be directly oven-dried and mounted with neutral balsam without the need for gradient alcohol dehydration. This approach yields more vivid, deep green staining results with a more pronounced contrast to DAB chromogen staining.

The hydrogen peroxide decolorization method utilizes the strong oxidative properties of hydrogen peroxide to remove melanin, aiming to reduce background interference from melanin granules. However, the use of strong oxidizers in the decolorization process can adversely affect tissue structure and antigen expression. Studies have shown that while hydrogen peroxide is effective at removing melanin, its strong oxidative nature can damage the tissue structure, leading to cellular and structural damage and even causing the detachment of tissue sections. This damage not only affects tissue integrity but also may interfere with antigen localization and expression^11,18^. In the hydrogen peroxide decolorization group in this study, some tissue sections presented residual melanin, weakened positive IHC intensity, tissue damage, and disruption of the cellular morphology and structure, with some sections showing detachment (Fig. 2F, I, L, and O). The tissue integrity score of the hydrogen peroxide decolorization group was significantly lower than that of the other groups.

The duration of hydrogen peroxide decolorization is related to the melanin content; the higher the melanin content is, the longer the decolorization time typically needed. This process may lead to incomplete or excessive decolorization, thereby affecting the accuracy of the staining results. Additionally, reports in the literature indicate that the impact of oxidizers on different antigens is selective, meaning that the same decolorization duration can have varying effects on different antigens^18^. This study also revealed that the expression intensity of Melan A was most affected, followed by that of HMB-45, with S-100 and Ki-67 also affected to varying degrees. The selectivity and unpredictability of these effects increase the complexity of the procedure, making the standardization and optimization of the oxidative decolorization method more challenging.

In the realm of melanin removal techniques, both strong oxidizing decolorization methods and differential staining approaches have their respective merits and limitations. While strong oxidizers are effective in melanin clearance, their potential to damage tissues and antigens, the uncertainty surrounding the decolorization process, and the complexity of their application restrict their utility. On the other hand, heterochromatic staining, such as the methylene blue restaining technique, overcomes many of the issues associated with strong oxidizers. These methods provide distinct color contrasts and simplified operations, enhancing the methodological framework for histological analysis.

This study has certain limitations, such as its small sample size and constraints in experimental conditions. Future research should aim to expand the sample size and delve into the specific chemical reaction mechanisms involved in hydrogen peroxide decolorization to optimize the method and mitigate tissue damage. Additionally, exploring a broader array of restaining agents and decolorizers, as well as developing more standardized and automated staining equipment, will contribute to enhancing the precision and reliability of melanin staining techniques. It is also possible to conduct multidimensional comparative studies with more traditional methods, such as potassium permanganate decolorization and azure blue restaining methods, to fully assess the effectiveness and value of methylene blue staining.

In summary, the methylene blue restaining method has notable advantages for melanoma IHC staining, including simplicity, time efficiency, and antigen preservation. While the hydrogen peroxide decolorization method effectively mitigates melanin background interference, its application is complicated by the risk of tissue and antigen damage, as well as procedural complexity, necessitating careful consideration in practical use. By meticulously evaluating the benefits and drawbacks of these methods, researchers can select the most appropriate techniques to ensure precise melanin staining and obtain reliable experimental results.

## Materials and methods

### Experimental materials

A total of 46 paraffin-embedded pathological samples from patients diagnosed with melanoma were collected from the Department of Pathology at Yichang Central People’s Hospital between January 2021 and August 2023. Each sample was reexamined by a pathologist to confirm the presence of melanin. All the samples were fixed in 10% neutral buffered formalin, dehydrated through a graded series of ethanol, vitrified with xylene, and embedded in paraffin. This study was approved by the Ethics Committee of Yichang Central People’s Hospital. As this was a retrospective study, an informed consent waiver was granted by the Ethics Committee for all the patients involved. We adhered to all relevant guidelines and regulations throughout the conduct of this study.

### Primary reagents

The methylene blue solution used in this experiment was purchased from Zhuhai Beso Biotechnology Co., Ltd. (ready-to-use). The Melan A mouse monoclonal antibody (clone number is A103, ready-to-use) was obtained from Henan Sainuo Biotechnology Co., Ltd. The HMB-45 (clone number is HMB-45, ready-to-use) and Ki-67 (clone number is MIB-1, ready-to-use) mouse monoclonal antibodies were sourced from Fuzhou Maixin Biotechnology Development Co., Ltd. The PRAME rabbit monoclonal antibody was procured from Abcam (clone number is EPR20330, diluted 1:2000). The HRP (horse radish peroxidase)-labeled goat anti-mouse/rabbit universal secondary antibody and DAB chromogenic reagent (ready-to-use) were obtained from Henan Sainuo Biotechnology Co., Ltd. .

### Experimental grouping

All the paraffin-embedded tissue blocks were sectioned into 15 consecutive slices with a thickness of 4 μm. These sections were mounted on adhesion slides in a uniform direction to prevent detachment and then incubated at 75 °C for 40 min. The baked tissue sections were divided into four groups. The first group consisted of 1 section, which underwent routine hematoxylin‒eosin (HE) staining and was designated the HE-stained group. The second group consisted of 5 sections, which were subjected to routine IHC staining followed by hematoxylin counterstaining, designated the routine IHC group (one of these sections was treated with PBS instead of the primary antibody as a negative control). The third group consisted of 5 sections, which were subjected to routine IHC staining followed by hematoxylin counterstaining and then counterstained with methylene blue, which was designated the Methylene Blue Restaining Group (one of these sections was treated with PBS instead of the primary antibody as a negative control). The fourth group consisted of 5 sections, which were first treated with hydrogen peroxide for melanin bleaching, followed by routine IHC staining and hematoxylin counterstaining; this group was designated the hydrogen peroxide decolorization group (one of these sections was treated with PBS instead of the primary antibody as a negative control).

### Experimental procedures

**Fig. 3:**
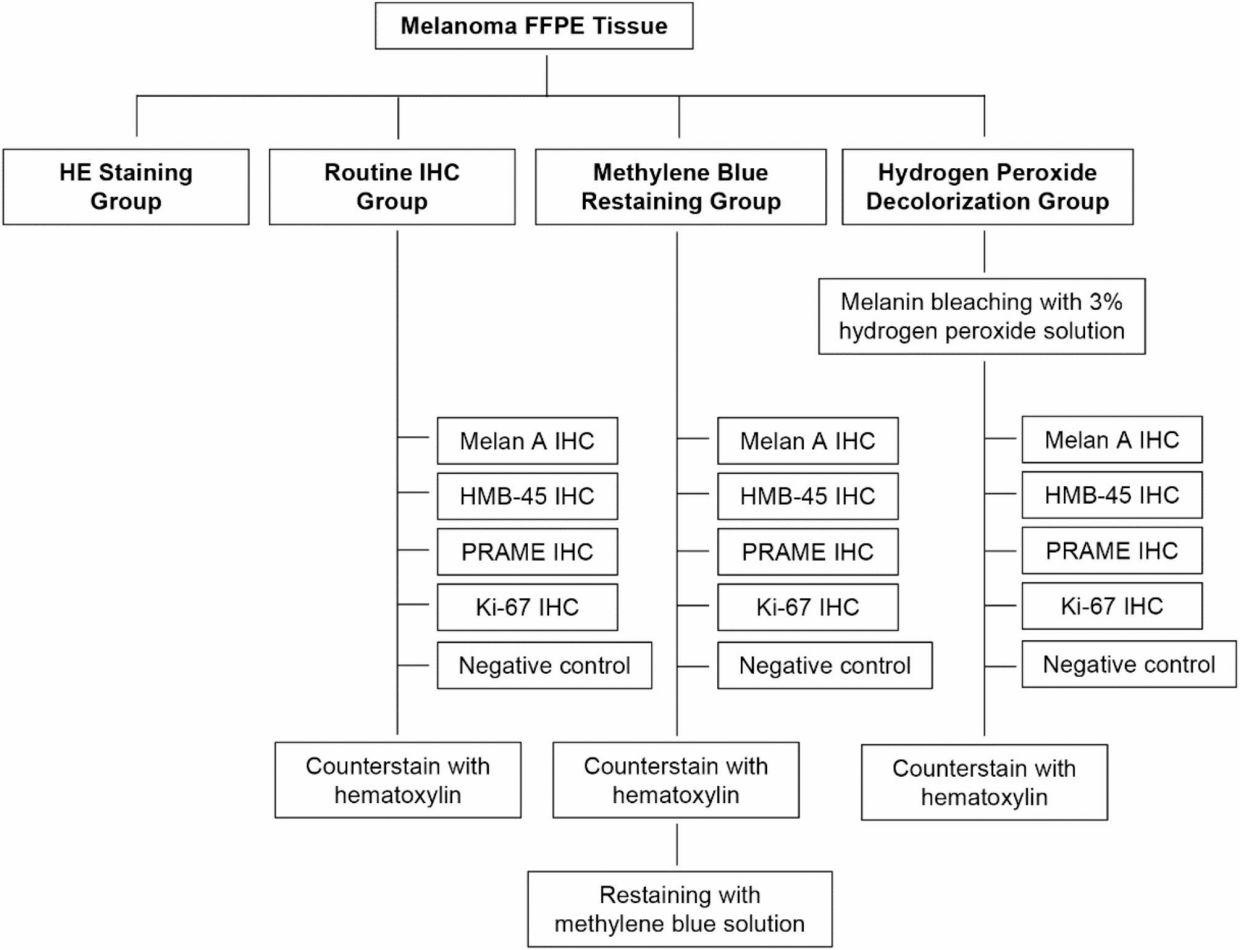
Schematic diagram of the experimental procedure.

A schematic diagram of the experimental design of the present study is shown in Fig. 3.

HE Staining Group: HE staining was performed on tissue sections using the automatic staining and coverslipping system (DAKEWE, China). Initially, the sections were deparaffinized in three tanks of xylene for 5 min each, followed by sequential passage through two tanks of anhydrous ethanol, one tank of 95% ethanol, and one tank of 85% ethanol, each for 3 min. The sections were then rinsed under running water for 3 min, stained in hematoxylin for 12 min, rinsed under running water for 1 min, and differentiated in 0.5% hydrochloric acid alcohol for 10 s. After rinsing under running water for 2 min, the sections were treated with EDTA blue solution (pH 9.0) for 2 min, rinsed under running water for 2 min, and then stained with eosin for 2 min. Finally, the sections were dehydrated through a graded series of alcohol and coverslipped with neutral resin.

Routine IHC Group: Tissue sections underwent the same deparaffinization process as described above. After rinsing under running water for 3 min, antigen retrieval was performed on the PT Module (Thermo Fisher Scientific, America) using EDTA (pH 9.0) at 98 °C for 20 min. Following retrieval, sections were incubated in a 3% hydrogen peroxide solution for 10 min to quench endogenous peroxidase activity. After delineation with a hydrophobic pen, sections were transferred to the AUTOSTAINER 720 (Thermo Fisher Scientific, America) for immunostaining with Melan A, HMB-45, PRAME, and Ki-67 antibodies, with a primary antibody incubation of 60 min, secondary antibody incubation of 30 min, and DAB development for 7 min. After color development, the sections were counterstained with hematoxylin and coverslipped on the DAKEWE automatic staining and coverslipping system, following the same procedure as described for the HE staining group.

Methyl Blue Counterstaining Group: The methyl blue counterstaining group followed the same procedures as the routine IHC group up to the blueing step. After blueing, the sections were removed from the instrument, manually treated with methyl blue solution for 3 s, thoroughly rinsed under running water, air-dried in a 60 °C oven, and then coverslipped with neutral resin.

Hydrogen Peroxide Bleaching Group: The hydrogen peroxide bleaching group followed the routine IHC group’s procedures with an additional step of treating with a 3% hydrogen peroxide solution to bleach melanin before antigen retrieval. The duration of melanin bleaching was adjusted based on the amount of melanin and the desired bleaching effect.

### Interpretation of results

All the tissue sections were independently reviewed and scored under a microscope by three associate chief physicians. The final score was determined by averaging the scores of the three reviewers. The scoring criterion was as follows: contrast between melanin and tumor cell staining: 2 points (0 points if indistinguishable, 1 point if contrast is poor but identifiable, 2 points if contrast is obvious). Clarity of staining: 2 points (0 points if unclear, 1 point if relatively clear, 2 points if clear). The degree of tissue completeness was 2 points (0 points if incomplete, 1 point if relatively complete, and 2 points if complete). A final score of 0 indicates that a diagnosis cannot be made, whereas a score of 6 signifies a complete diagnosis.

### Statistical analysis

All the data were statistically analyzed via SPSS version 27.0. Paired *t* tests were employed to compare the results between the experimental and control groups. A *P* value of less than 0.05 was considered statistically significant.

## Data Availability

All data generated or analysed during this study are included in this published article and its supplementary information files.
